# Experimental and probabilistic model validation of ultrasonic MEMS transceiver for blood glucose sensing

**DOI:** 10.1038/s41598-022-25717-x

**Published:** 2022-12-08

**Authors:** Hara Prasada Tripathy, Priyabrata Pattanaik, Dilip Kumar Mishra, Sushanta Kumar Kamilla, William Holderbaum

**Affiliations:** 1 Semiconductor Research Laboratory, Faculty of Engineering and Technology (ITER), Siksha ‘O’ Anusandhan (Deemed to be University), Bhubaneswar, 751030 India; 2 School of Biological Science, Biomedical Engineering, University of Reading, Whiteknights, RG6 6AY UK

**Keywords:** Electrical and electronic engineering, Actuators, Sensors and biosensors

## Abstract

In contrast to traditional laboratory glucose monitoring, recent developments have focused on blood glucose self-monitoring and providing patients with a self-monitoring device. This paper proposes a system based on ultrasound principles for quantifying glucose levels in blood by conducting an in-vitro experiment with goat blood before human blood. The ultrasonic transceiver is powered by a frequency generator that operates at 40 kHz and 1.6 V, and variations in glucose level affect the ultrasonic transceiver readings. The RVM probabilistic model is used to determine the variation in glucose levels in a blood sample. Blood glucose levels are measured simultaneously using a commercial glucose metre for confirmation. The experimental data values proposed are highly correlated with commercial glucose metre readings. The proposed ultrasonic MEMS-based blood glucometer measures a glucose level of $257\pm 21$ mg/dl. In the near future, the miniature version of the experimental model may be useful to human society.

## Introduction

Chronic disease diabetes, commonly known as ‘Diabetes Mellitus (DM)’ is increasing exponentially and expected to exceed 552 million patients by 2030. This is the prediction of world health organization (WHO)^1,2^. This causes the mortality of age group from 20 to 70 years. Due to this growing incident, the WHO has declared the DM as a world epidemic. Precaution is better than cure and cure in the case of diabetes is a critical aspect to achieve. The global health-care expenditure on diabetes management has annually expected to be exceeded from 376 billion USD to 490 billion USD by 2030^3^. Hence, precaution can be possibly taken by frequent glucose monitoring and maintaining the physical glucose level for which it is necessary to fabricate/design the best glucose monitoring system having low-cost, simple fabrication process, continuous monitoring and robust.

The commercial non-invasive or minimally invasive glucose meters generally quantify the glucose levels from the blood samples. These methods are expensive due to high-cost test strips and painful each time when pricking into fingertip is conducted for taking blood samples. Non-invasive glucose meters are being researched for continuous painless measurement in an ongoing way^4^. The present non-invasive glucose monitoring methods are the fluorescence, the transdermal extraction, the electromagnetic variations, the polarimetry, the spectroscopy^5–8^. The accuracy and cost of these methods are the prime concern including method simplicity. The availed commercial blood glucose meters (BGM) reach an accuracy of 96%^9,10^. To achieve better accuracy more than the adopted range, new methods are on the verse of searching. These methods are mostly based on non-invasive systems. Some of the combined techniques are present for the quantification of glucose from glucose solutions^11^ and also from intralipid phantom medium^12^.

The goal of many developers and researchers worldwide is to create non-invasive blood glucose monitoring technologies that will improve the lives of millions of individuals with diabetes. However, it is challenging to make these procedures as accurate as conventional test strips. The Google Contact Lens is one cutting-edge device whose development has terminated in 2018 because its approach of sensing glucose levels in tears is not reliable enough^13^.The German company DiaMonTech created the D-Base blood sugar monitor, which is the size of a shoebox. By passing an infrared laser through the skin of a finger and forcing the glucose in the skin to convert the light to heat, the device detects blood sugar levels. The system then determines the glucose levels based on how much the skin heats up, however the increase in temperature is too slight for the person to detect. D-Base is permitted for use by medical professionals in clinical trials and diabetic centres in the EU as of 2019. The business is now creating D-Base for the US market. Additionally, DiaMonTech is developing scaled-down versions of the technology, such as the D-Pocket, a handheld gadget, and a tiny sensor for wearable devices^14^.Eversense is a subcutaneous implant that continuously measures blood glucose levels and has created by the US business senseonics. Although a doctor must first implant the sensor beneath the skin, it can last for up to three months before needing to be replaced. Using a polymer that fluoresces in reaction to blood sugar levels, Eversense detects glucose in the interstitial fluid beneath the skin of the upper arm. The transmitter uses the data to show the blood glucose levels in real time^15^. Integrity Applications, a US-Israeli business, developed GlucoTrack, a device that uses a combination of ultrasonic, electromagnetic, and thermal waves to monitor blood sugar levels. To provide a reading, the sensor is attached to the ear. The device targets people with type 2 diabetes and is marketed in Europe. In order to enter the US market, Integrity Applications is developing the second iteration of GlucoTrack, which consists of a wireless ear clip sensor connected to a smartphone^16^. A sensor called GlucoWise is currently being developed that could check blood sugar levels by just touching the skin between the thumb and forefinger. A smartphone app might then receive the real-time measurements. According to the designers, the device ought to be more precise than previous wireless glucose monitors because it measures blood glucose levels using a particular frequency of radio waves^17^. An under eyelid blood sugar monitor that can wirelessly transmit glucose readings to a smartphone is being developed by a Dutch business called NovioSense. A flexible metal coil with nanosensors inside that is only two centimetres long makes up the device. A soft hydrogel layer of protection is then placed over the coil. Using the same enzyme technology as standard glucose strip testing, the coil could measure minute variations in the glucose levels of tear fluid. The device’s accuracy is comparable to the FreeStyle Libre, according to findings from a clinical research that has released last year^17^. The phrase “the eyes are windows to the soul” is taken literally by Occuity Indigo as an eye-focused blood glucose metre. The UK developer Occuity instead examines within the eyeball since it is a transparent, stable environment whose glucose levels correlate with those of the blood, as opposed to detecting tear fluid, as did the Google Contact Lens. The Occuity Indigo measures the light that reflects back into the gadget after sending a weak beam of light into the eye. Based on the refraction of the light returned, it can deduce the amount of glucose present in the eye^18^. A changeable skin patch with a transmitter is called SugarBEAT, and it was created by the British biotech company Nemaura Medical. By applying a little electric current to the skin and drawing out a sample of the interstitial fluid, which is located immediately below the skin, it monitors blood glucose levels non-invasively. Every 5 mins, the rechargeable transmitter delivers data over Bluetooth to the user’s phone, where the readings may be seen using a companion app^19^.

Several attempts have been undertaken in optical techniques using visible, near, and mid infrared (NIR and MIR) light^20,21^ and as well as in non-optical techniques to measure the blood glucose density. However, the scattering phenomenon and the lower bandwidth has a poor correlation with glucose in the blood. Again, the fundamental limitation of MIR is that it can only be used in reflectance mode because of limited penetration depth in the human tissue for which it is unable to estimate glucose concentrations in blood vessels. The considerable scattering of light and other physiological factors limits the use of optical polarimetry for the measurement of blood glucose through skin^22,23^. Microwave devices, particularly planar resonant ones, have several advantages, such as simplicity of fabrication and integration, low dimension, competitive cost, and intriguing penetration depths, particularly for non-invasive measurements with minimal tissue scattering^24,25^. The fundamentals and convenience of planar resonant based measurements are deeply studied^26^ in an operating frequency range from 0.64 to 18.63 GHz and designed 3D printable GHz ranged devices^27^ on account of glucose sensing. However, the only disadvantage of using the high frequency range in GHz region is that it creates damage to tissues if used continuously for a prolonged period to sense the glucose in non-invasive way. For which in our research work, we have chosen low frequency range in the order of kHz. So our proposed research work is carried out at 40 kHz ultrasonic MEMS transceiver for blood glucose sensing. Ultrasonic technique is used as an alternate to microwave technique. WZT40 and WZR40 pair is a low powered unidirectional ultrasonic transceiver procured from $electronics-store4u$ with a sensitivity of $-63\pm -3 {\text{dB}}$ at 40 kHz Sine wave. The recent focus is to design hassle free painless device to measure the blood glucose. But all the devices are in developing stage and thus the rectification including the clinical trials are in continuous process^28^.

Insulin resistance is linked to an increase in serum albumin levels. However, serum albumin had no influence on the development of diabetes on its own. Although extremely low quantities of protein in urine are normal, large levels of albumin are one of numerous markers of chronic kidney disease (CKD), a frequent consequence of both type 1 and type 2 diabetes^29^. Previously, ultrasonic principle-based MEMS glucose sensors based on various bio-friendly piezoelectric materials were proposed^30,31^. Prior to conducting experimental practises, simulations are performed using leading piezoelectric materials to test the principle’s reliability^32^. Transceivers based on piezoelectric materials are used to generate and receive directional ultrasonic waves that are passed through the blood sample medium. With the commercial blood glucose metre, the received signal quantifies the glucose level of the blood medium in a simulation environment and achieves an accuracy level of 99% (CBGM)^33^.In the research, we have considered immovable blood obtained from healthy goats for our experiment in a testing beaker and have focused to track the glucose density only. The concentration of some proteins rather than glucose in blood changes, which may change the blood density so as the measurement and thus limits the device measurement. The current work is a proposed experimental system to mimic the previous simulative approach^33^ with the addition of a probabilistic model for the quantification of the result values into glucose level by mapping CBGM data values. The rest of this research paper is organised as follows: Section II describes the experimental system setup. Section III covered the probabilistic model analysis with error grid analysis. Section IV illustrates the results and discussion of the proposed system, and Section V summarises the significance of our research.

**Figure 1:**
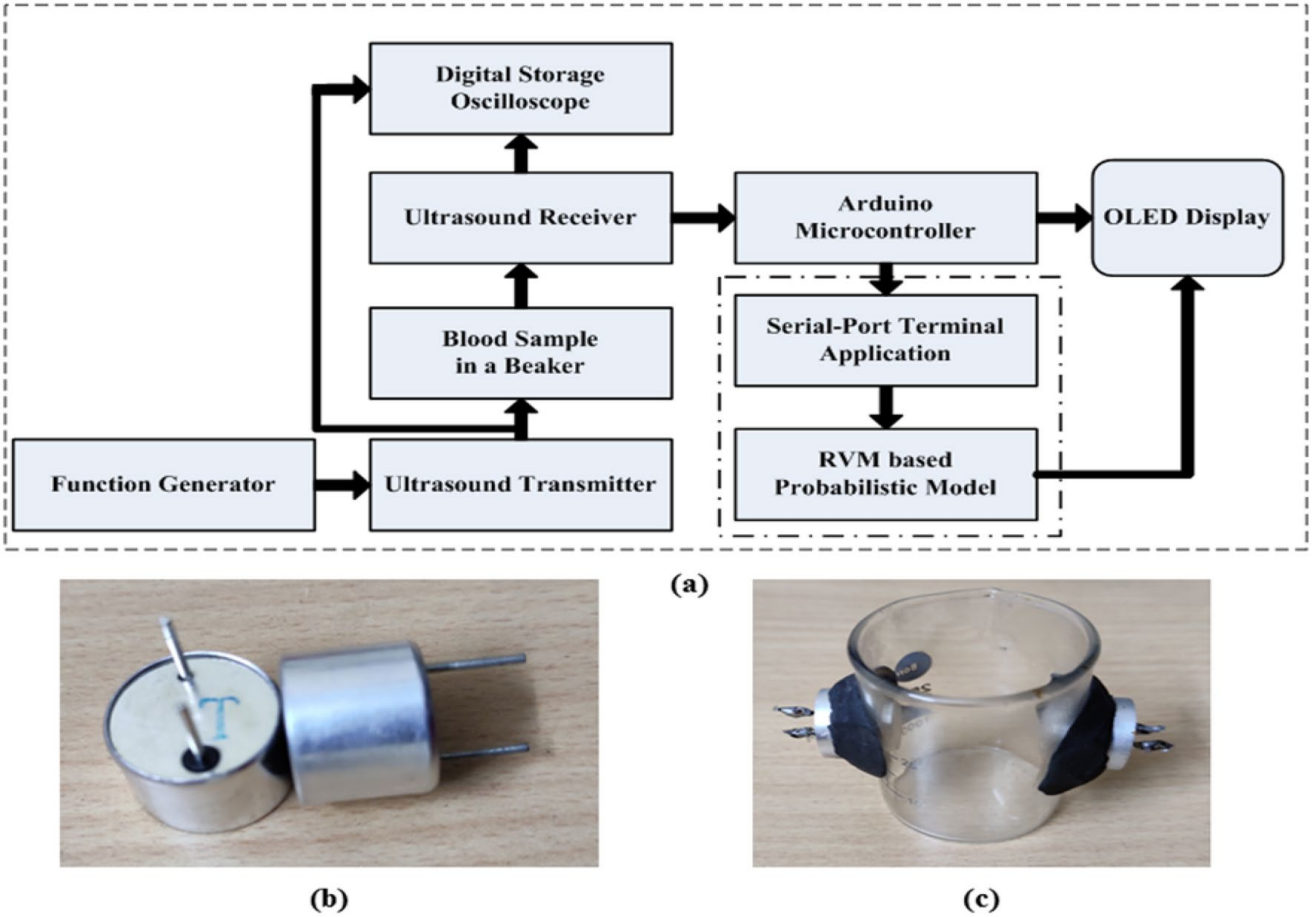
(**a**) Flowchart representation of blood glucose level measurement setup; (**b**) Procured ultrasonic transceiver (WZT40 and WZR40) pair; and (**c**) Sample holder with ultrasonic transceiver (WZT40 and WZR40) pair.

## Experimental system setup

This article section proposes an experimental discourse of designing a system based on an ultrasonic MEMS transceiver setup for blood glucose level sensing. Flow chat representation of the blood glucose level measurement approach can be clearly seen from the Fig. 1a. The work flow process consists of a pair of ultrasonic transceiver (WZT40 and WZR40), sample holding glass beaker (10 ml), a function generator (SDG1025, 25 MHz, 125 MSa/s Sample Rate), digital storage oscilloscope (Scientech 403, 100 MHz, 1 GSa/s), Arduino micro-controller kit, OLED Display with SSD1306 Driver IC (Geekcreit 0.91 Inch 128 × 32 IIC I2C) and RVM model. A pair of ultrasonic transceiver (WZT40 and WZR40) (Fig 1b) is procured from market and thus affixed in the glass beaker by making a circular cut on the wall. The sample holder (beaker) with ultrasonic transceiver is represented in Fig. 1c. Blood density is related to hematocrit, or more precisely, to total protein content in blood. Osmotic pressure, immunity, and coagulation of blood fluctuate due to the variation in proteins^29^. However, other plasma solutes like sodium, potassium, chloride, bicarbonate, magnesium, calcium amino acids, vitamins, organic acids, pigments, and enzymes have only a minimal impact on blood density, which is not given importance in our study. In the research, we have considered immovable blood obtained from healthy goats for our experiment in a testing beaker and have focused to track the glucose density only.

In our study, we have considered the animal blood. The choice of the animal blood is undertaken as there is no difference in composition of the blood in consideration to mammals and human. However, the percentage of protein is different by which any two of the species can be identified from each other. Blood samples are collected in blood collection tubes (IVD, VAKU-8) from a registered slaughter house. The collection tube contains additives like sodium fluoride and ethylene-diamine-tetra-acetic acid (NaF+EDTA). The additive is an anticoagulation agent intended to use for glucose detection. The blood medium is considered as a medium of ultrasonic wave propagation. To generate an ultrasonic wave, a function generator of optimized frequency 40 kHz and 1.6 V voltage peak to peak (vpp) is applied. A digital storage oscilloscope is used to trace the voltage values from the ultrasonic receiver. The variation in glucose levels (0–450 mg/dl) in the blood medium is traced distinctly from the receiving voltage values.

For commercial data value reading on parallel with the proposed method of blood glucose sensing, an Accu-check Aviva glucometer is used. When an ACCU-CHEK®Aviva Plus test strip is inserted into the ACCU-CHEK®Aviva meter, a small alternating current (AC) is applied until the application of blood on the test strip causes a closed circuit between both the standard and measurement electrodes. Both are used to assure an adequate sample has been applied. The meter then applies a series of AC voltages at four frequencies and reads the AC responses. These carry information about the sample type and environmental temperature; they also allow the system to perform various internal quality checks. After the AC measures are completed, a small (DC) voltage is applied and current is observed which is proportionate to the glucose. The AC and DC information are then combined to provide a hematocrit and temperature compensated glucose result. The enzyme on the test strip, a variant of glucose dehydrogenase, converts the glucose in the blood sample to gluconolactone. This reaction creates a harmless DC electrical current that the meter interprets for the blood glucose result. The sample and environmental conditions are also evaluated using small AC signal.

The entire observation is carried out in a controlled environmental condition. The Arduino micro-controller kit with the serial-port terminal application (coolTerm 1.5.0.572) is used to store the receiving voltage values from the receiver continuously. For quantification, RVM model is used and result is displayed on the OLED display.

## Probabilistic model and error grid analysis

### Prediction analysis

**Figure 2:**
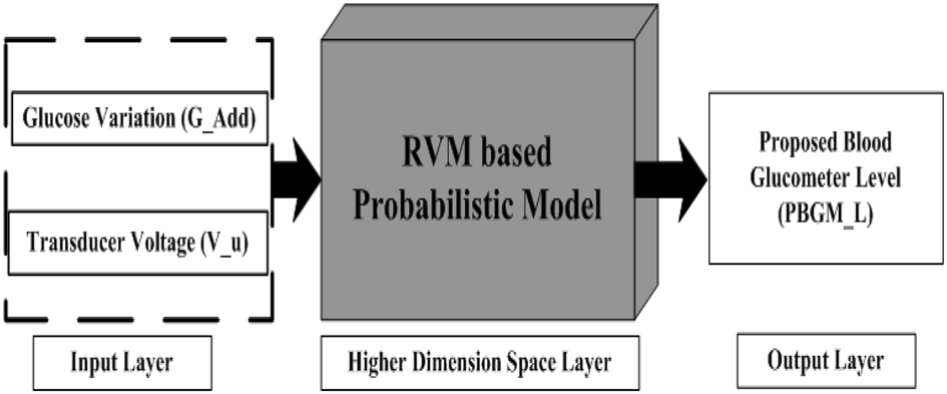
Schematic diagram of the RVM based prediction analysis.

The probabilistic model in which the output is evaluated based on the Bayesian paradigm and the weights as hyper-priors is termed as RVM. As like our previous work^34^,we have implemented RVM probabilistic model to predict glucose level from added glucose variation and ultrasonic transducer data. RVM based block diagram is depicted in Fig. 2. The model is designed in such a way, that the model takes input parameters as the added glucose variation ($G_{Add}$), the ultrasonic transducer voltage output ($v_u$) and gives rise to the output which is proposed blood glucose meter level ($PBGM_L$).

The RVM model consists of three layers such as the layer-1 (input layer feature vector for *i*th instance, layer-2 (hidden layer or higher dimension space layer) and layer-3 (output layer with the predicted blood glucose values). The input layer *i*th instance is denoted as $$\textbf{m}_{i}=\begin{bmatrix} G_{Add}&v_{u} \end{bmatrix}$$). The RVM model is used to predict glucose level from added glucose variation $\tilde{G_Add}$ and ultrasonic transducer data $\tilde{v_u}$. In the model,$\phi$ corresponds to the design matrix and the K specifies the kernel function which maps the input features to high dimensional feature space just alike as SVM. The output layer which is layer 3 and it generates the glucose level named as proposed glucose meter reading PBGML respectively. To evaluate glucose level, the concerned mathematical formulation of the RVM model is as follows

1 $$\begin{aligned} \tilde{PBGM_L}=PBGM_L(\mathbf {m_{i}}, w)+\epsilon _{i} \end{aligned}$$

where the actual and predicted glucose levels for *i*th exemplar are $\tilde{PBGM_L}$ and $PBGM_L(\mathbf {m_{i}}, \textbf{w})$. The glucose level data values for the model are the values, which have been taken simultaneously at the time of experiments by accu-check instant (commercial glucometer). The weight vector between the layer-2 and layer-3 of probabilistic model is $$\textbf{s}=\begin{bmatrix} s_{1},&s_{2},&\ldots s_{N} \end{bmatrix}^T$$. The noise of the zero-mean Gaussian process with a variance of $\sigma ^2$^35^ termed as $\varepsilon _{i}$ in Eq. (1). Similarly, the training set is evaluted by using the below given Eq. (2)^36^.

2 $$\begin{aligned} P(\tilde{PBGM_L}\mid \textbf{w},\sigma ^{2})&= \prod _{i=1}^{N}P({PBGM_L}_{i} \mid \textbf{w},\sigma ^{2}) \nonumber \\&= \prod _{i=1}^{N}{\frac{1}{(2\pi \sigma ^{2})^\frac{N}{2}}}exp\left\{ -\frac{1}{2\sigma ^{2}}\parallel {PBGM_L}_{i}-\phi (\textbf{m}_{i}) \textbf{w}\parallel ^{2} \right\} \end{aligned}$$

The $\phi$ tends to the design matrix. This has termed as $\phi =[\phi (\textbf{m}_{1}),\phi (\textbf{m}_{2}), \ldots \phi (\textbf{m}_{N})]^T$. The non-linear basis function for *i*th exemplar is $\phi (\textbf{m}_{i})$ and evaluated by the kernel function $\phi (\textbf{m}_{i})=[1,K(\textbf{m},\textbf{m}_{1}),K(\textbf{m},\textbf{m}_{2}), \ldots ,k(\textbf{m},\textbf{m}_{N})]$. The prior weight distribution for the RVM model is $P\left( w \mid a \right) = \prod _{i=1}^{N}N\left( w_i \mid 0, \alpha _i^{-1} \right)$, where w is weight vector between layer-2 and 3 of the model and is a set of hyperparameter. In order to avoid the over fitting in the estimation of *w* and $\sigma ^2$, a set of hyperparameter has been introduced in RVM. This means the weight vector is sparse and thus, the RVM model is named as sparse Bayesian model. The posterior probability distribution $P\left( w,\alpha , \sigma ^2 \mid \widetilde{PBGM_L} \right)$ can be evaluated as $P\left( w,\alpha , \sigma ^2 \mid \widetilde{PBGM_L} \right) =P\left( w \mid \widetilde{PBGM_L},\alpha , \sigma ^2 \right) \times P\left( \alpha , \sigma ^2 \mid \widetilde{PBGM_L}\right)$. The first term of the posterior distribution over weight is computed as Eq. (3)

3 $$\begin{aligned} P\left( w \mid \widetilde{PBGM_L},\alpha , \sigma ^2 \right) =2\pi ^{-\frac{(N+1)}{2}}C^{-\frac{1}{2}}\times \exp \left( -\frac{1}{2}(w-\mu ^{-1})C^{-1}(w-\mu )\right) \end{aligned}$$

The mean and convergence matrix computed from the above equation are given by $C=(\sigma ^{-2}\acute{\phi }\phi +A)^{-1}$ and $\mu =\sigma ^{-2}C\phi \widetilde{PBGM_L}$, respectively. Where A, is a diagonal matrix with entries as $(\alpha _{0},\alpha _{1} \ldots \alpha _{N})$. The second term of the posterior equation can be written as, $P\left( \alpha , \sigma ^2 \mid \widetilde{PBGM_L}\right) =P\left( \widetilde{PBGM_L}\mid \alpha , \sigma ^2\right) P\left( a\right) P\left( \sigma ^2\right)$. Thus, the distribution can be evaluated by Eq. (5) as

4 $$\begin{aligned} P\left( \alpha , \sigma ^2 \mid \widetilde{PBGM_L}\right)&=P\left( \widetilde{PBGM_L}\mid \alpha , \sigma ^2\right) P\left( {w \mid a }\right) \end{aligned}$$

5 $$\begin{aligned} P\left( \alpha , \sigma ^2 \mid \widetilde{PBGM_L}\right)&=2\pi ^{-\frac{N}{2}}\left( \sigma ^{2}I+\phi A^{-1}\acute{\phi } \right) ^{-\frac{1}{2}}\times \exp \left( -\frac{1}{2}\acute{\widetilde{PBGM_L}}\left( \sigma ^{2}I+\phi A^{-1}\acute{\phi } \right) ^{-1} \widetilde{PBGM_L}\right) \end{aligned}$$

The iterative estimation of the hyperparameter and variance are given by $\alpha _{i}^{new}=\frac{\gamma _i }{\mu _{i}^{2}}$ and $(\sigma ^{2})^{new}=\frac{\parallel {\acute{\widetilde{PBGM_L}}}-\mu \phi \parallel }{N-\sum _{i}\gamma _{i}}$i ), where $\mu _{i}$ corresponds to *i*th posterior average weight and$\gamma _{i}=1-\alpha _{i}C_{ii}$. The $C_{ii}$ is the diagonal element of posterior covariance matrix. The RVM prediction for a new feature vector $z_{t}^{*}$ is given by Eq. (6)

6 $$\begin{aligned} P\left( \widetilde{PBGM_L}\mid z_{t}^{*},\alpha _{mp}, \sigma _{mp}^2 \right) =\int P\left( \widetilde{PBGM_L}\mid w,\sigma _{mp}^2 \right) P\left( w\mid \alpha _{mp}, \sigma _{mp}^2 \right) dw \end{aligned}$$

where optimal parameters such as $\alpha _{mp}$ and $\sigma _{mp}^2$ are computed iteratively using the hyper-parameter and variance. In order to avoid the over-fitting in the estimation of $\textbf{w}$ and $\sigma ^2$, a set of hyperparameter $(\alpha )$ has been introduced in RVM which means the weight vector is sparse and this RVM model also called as the sparse Bayesian model^37^. After the data prediction from RVM model, the normalized correlation coefficient (NCC) is used to estimate the similarity with CBGM data values.

### Error grid analysis between CBGM and PBGM

#### Clarke‘s error grid analysis

The delimitation between CBGM and PBGM is a much needed analysis for medical importance. The Clarke error grid analysis provides a platform for blood glucose meter (BGM) developers inducing researchers working in this domain. Though the analytical model provided by Clarke et al.^38,39^ release 35 years (in 1987) back but still it has merit and adopted widely. This approach includes a graphical cartesian plot with designated nomenclatures for the identification of glucose levels. For identification of blood glucose level, if CBGM value is 120 mg/dL where PBGM value 123 mg/dL then the graph denotes for this particular level value as (120,123) in the XY cartesian domain. The clarke‘s error grid has five specific zones which denote from A to E. The zones are denotes as the clinically correct decisions (Zone:A), the clinically uncritical decisions (Zone:B), overcorrection (Zone:C), skip a necessary correction (Zone:D) and performing the opposite/wrong correction respectively (Zone:E). This analysis does not specify the type of diabetes (type-1 or 2) and direct transition from zone B to E skipping C and D zones is not possible. Despite of wide acceptance and usefulness of this analysis, it carries the above limitations/criticisms.

**Figure 3:**
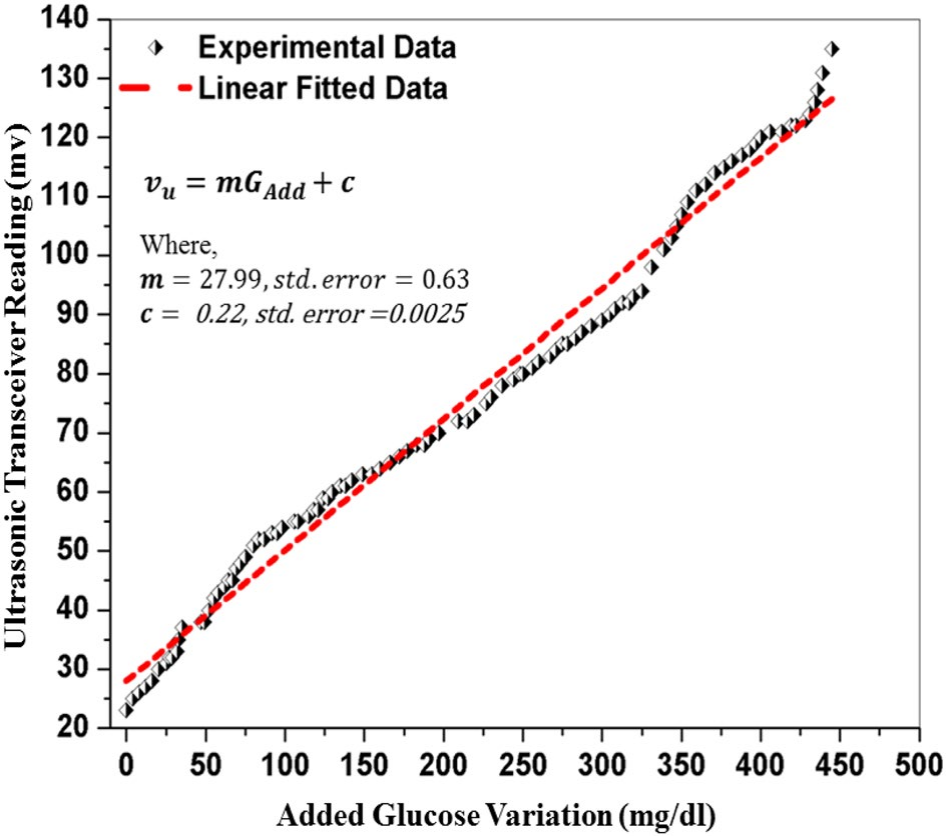
The variation in the receiving ultrasonic transceiver readings due to the fluctuation in blood glucose level.

#### Parke‘s error grid analysis

The drawbacks of Clarke’s error grid analysis have been solved in Parke‘s error grid analysis. Parke‘s error grid analysis tool is developed in June 1994, within 1 year after the publication of the Diabetes Control and Complications Trial study (DCCTS). The insulin therapy has not been widely accepted at that time so as analog insulins were not available^40^. The continuous transitions between zones, differentiation between type-1 and type-2 diabetes and the range from 0 to 550 mg/dl level are present in Parke‘s error grid analysis with no lower E zone.

#### Surveillance error grid analysis

The surveillance error grid (SEG) analysis is a tool for analysis and visualization of BGMs errors and has been certified by 206 diabetes clinicians and 28 non-clinicians for 4 different rated treatment scenarios^41^. The SEG is a tool to assess the degree of clinical risk from inaccurate BGMs. This is developed by Diabetes Technology Society (DTS) together with representatives from the Food and Drug Administration (FDA), the American Diabetes Association, the Endocrine Society, and the Association for the Advancement of Medical Instrumentation (AAMI), and representatives of academia, industry, and government.

**Figure 4:**
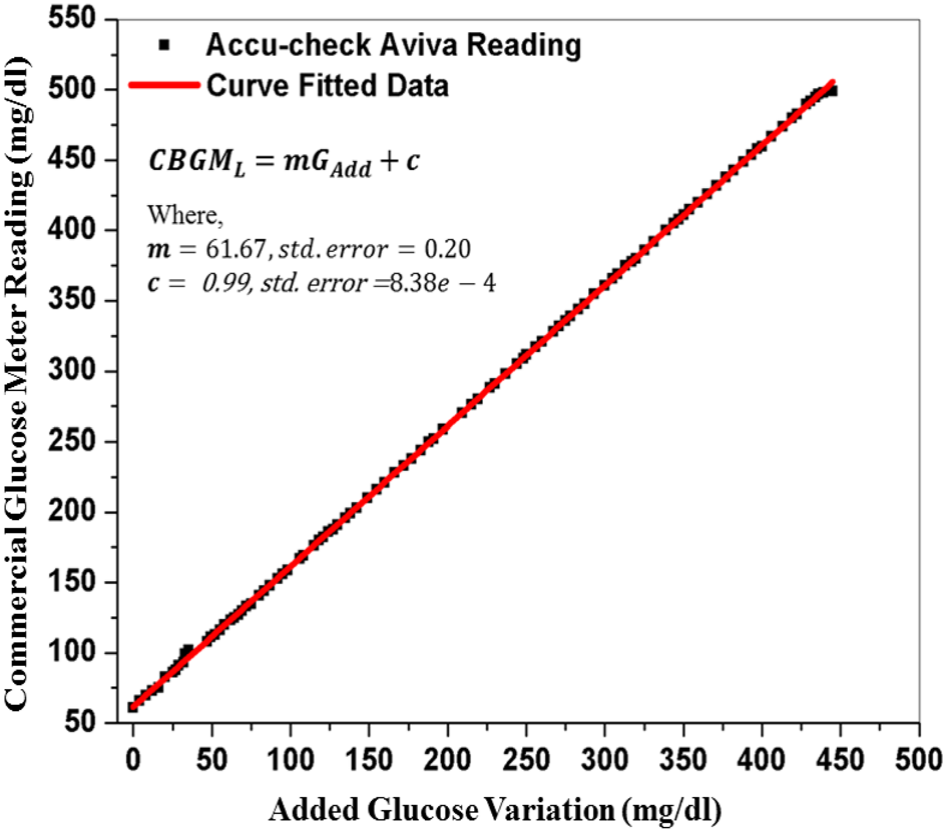
Mapping between the glucose variation in blood and the commercial glucose meter readings.

**Figure 5:**
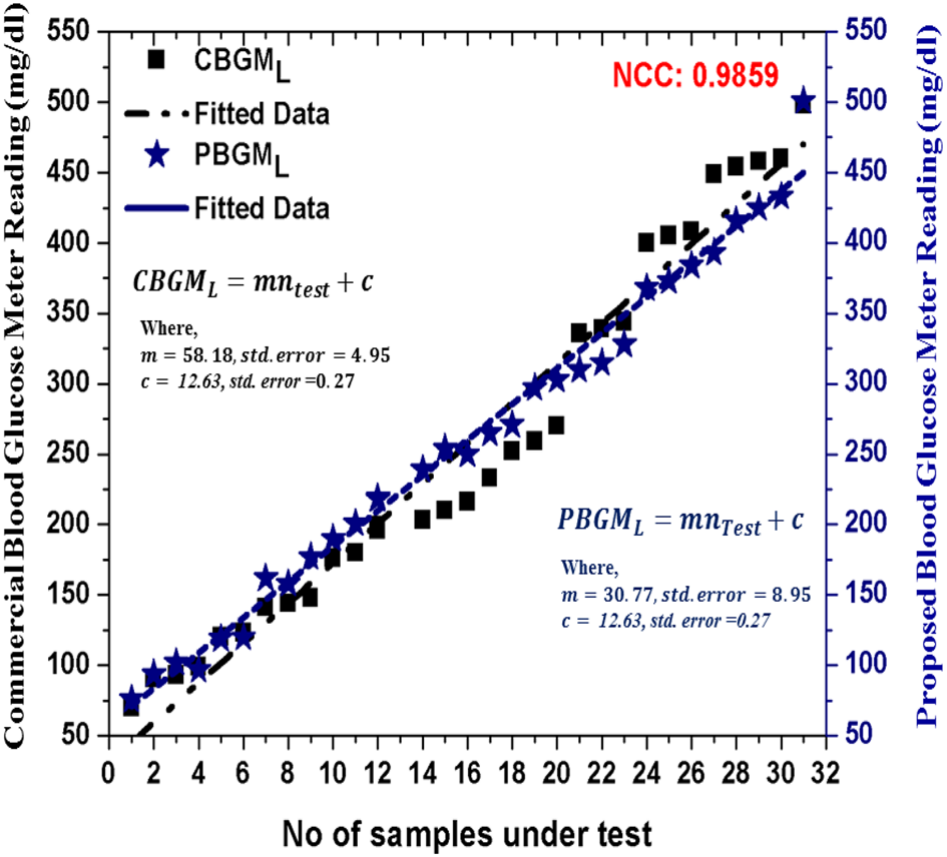
Corroboration between commercial and proposed blood glucose meter level.

## Experimental results with model validation and discussion

**Figure 6:**
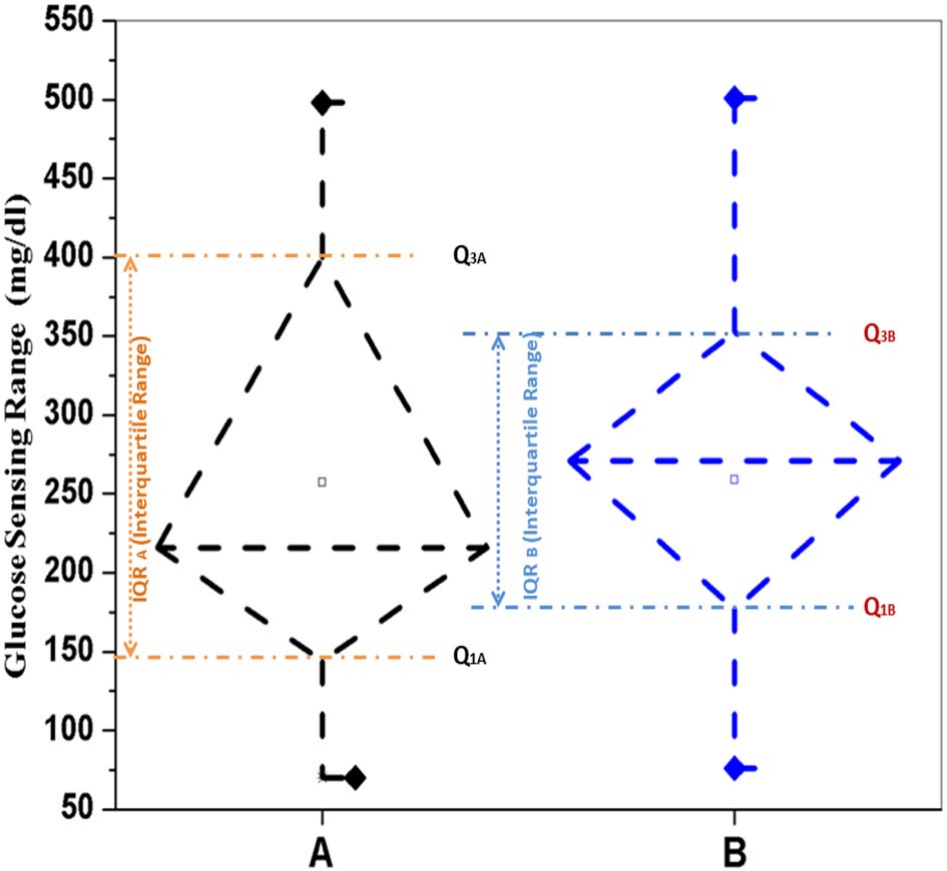
Glucose measured by (A) Accu-ckeck Aviva commercial glucose meter ($259.23\pm 109.37 {\text{mg/dl}}$) and (B) Proposed glucose meter ($257.23\pm 131.33 {\text{mg/dl}}$) (no statistically difference with $p>0.05$).

In this section, the glucose level data values obtained from the PBGM and CBGM at different glucose concentrations in the blood medium are shown. In the blood sample, glucose has been added 4 mg/dl at each batch manually and accordingly, the transceiver reading has been taken to observe the variation. In the same time, the commercial glucometer is used to take the readings. The correlation between glucose level values of the PBGM and the CBGM are depicted in Figs. 3 and 4. To quantify the glucose levels in the proposed system, the ultrasonic readings pass through the RVM probabilistic model. Figure 5 depicts the corroboration between $CBGM_L$ and $PBGM_L$. In the Fig. 5, we have taken the tested data sets after rigorous training of the RVM probabilistic model. All the data sets are trained by the model randomly to generate proposed glucose levels from the inputs (transducer voltage and glucose variation). After the model has been trained, it generates testing data sets. The no of testing data sets are 32 and they depicted in Fig. 5. This clearly indicates that the RVM model is able to quantify the glucose level inside the blood sample. The nonlinear correlation coefficient of 0.9859 with the commercial blood glucose meter level depicts the probabilistic ability of the model. It is evident that the glucose level values evaluated from PBGM are well correlated with the CBGM with a NCC value of 0.9859.

**Figure 7:**
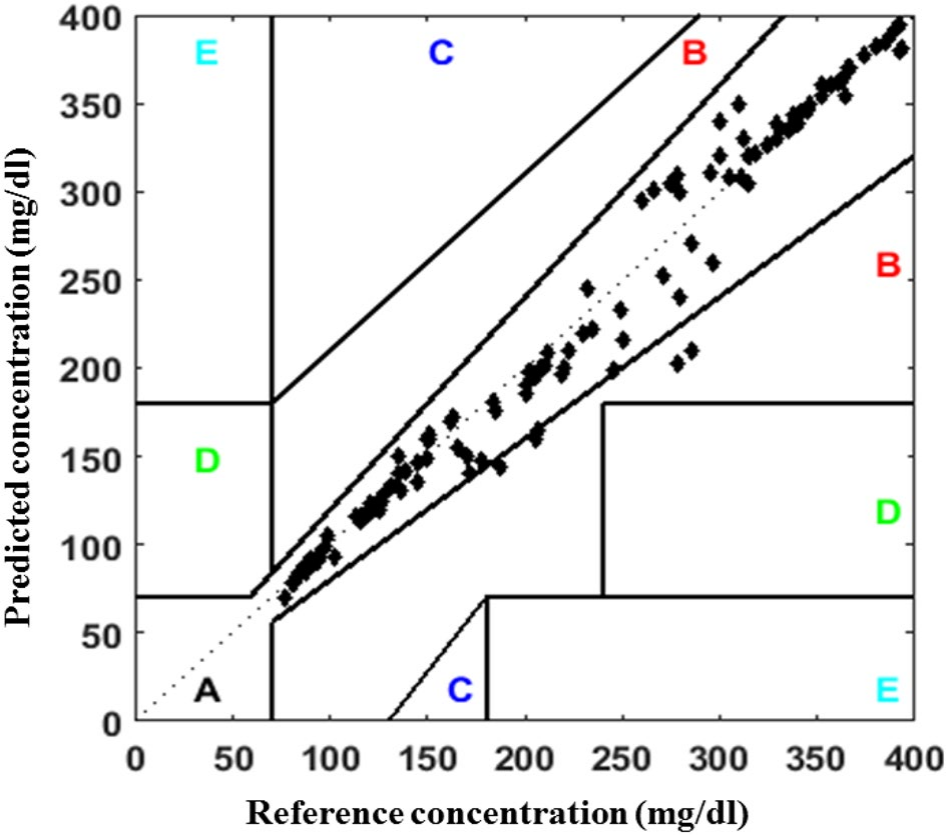
Clarke‘s error grid analysis for PBGM.

**Figure 8:**
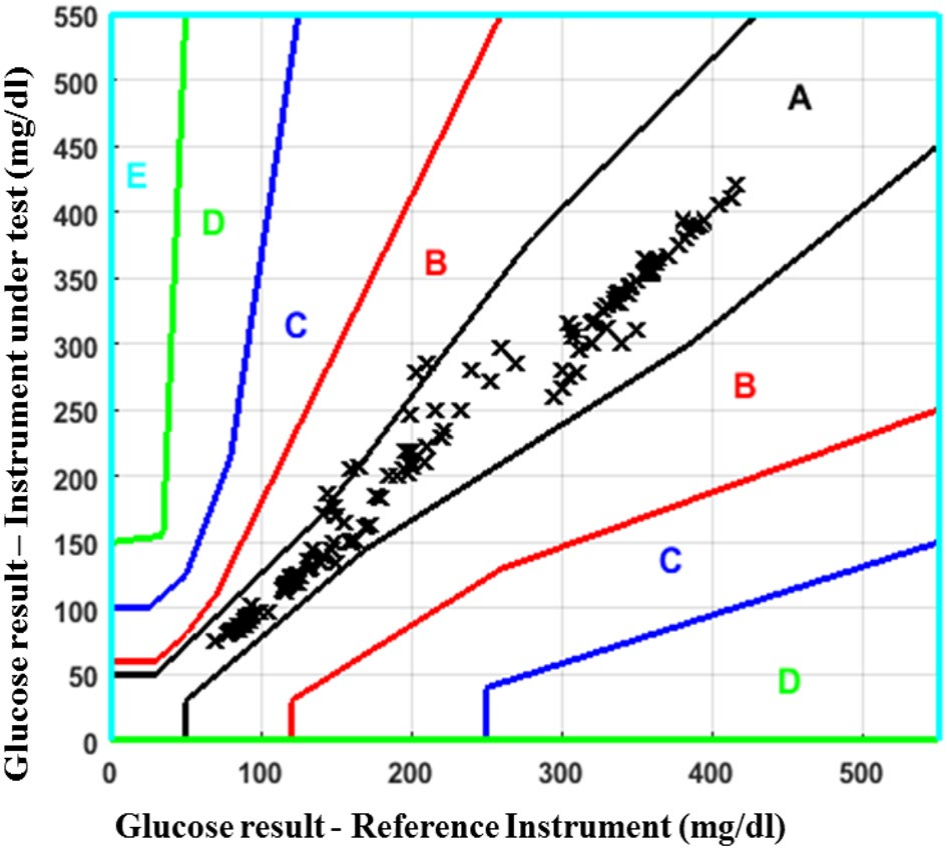
Parke‘s error grid analysis for PBGM.

The observations from the previous works reveal that the proposed ultrasonic MEMS based transceiver has the ability to senses the glucose levels in the blood medium and is well correlated with the glucometer readings. The blood glucose level determined by electrochemical glucose sensor is $356.0\pm 116 {\text{mg/dl}}$, and the glucose level quantify by the commercial blood glucometer is $424.8\pm 59 {\text{mg/dl}}$. The comparison of these two earlier reported devices have no statistically significant difference (p value) greater than 0.05^42^. However, the average blood glucose level determined by proposed simulated result is $280.4\pm 1450 {\text{mg/dl}}$, and the glucose level measured by the commercial glucose meter is $282.3\pm 149 {\text{mg/dl}}$^33^. The glucose level measured by $PBGM_L$ and $CBGM_L$ are $257.2\pm 131 {\text{mg/dl}}$ and $259.2\pm 109 {\text{mg/dl}}$ respectively. The box-plot between CBGM and PBGM is depicted in Fig. 6. A boxplot is a standardised method of depicting data distribution based on a five-number summary (“minimum, first quartile [Q1], median, third quartile [Q3], and maximum”). It can provide information about your outliers and their values. B boxplot refers to the symmetric structure and closely packed data. Because it is unaffected by outliers, the IQR (Interquartile Range) is typically regarded as a better measure of dispersion than the range. We utilized our data sets in the study to examine their distribution throughout sensing range, and it is symmetrical. A significant standard deviation shows that the observed data is very variable around the mean. In Fig. 6, we present the first quartile, second quartile, and interquartile range for Accu-check Aviva similarly and suggested blood glucose meter as Q1A, Q3A, IQR A and Q1B, Q3B, and IQR B. The comparison of above two devices shows no statistically significant difference (p value > 0.05). Figure 7 denotes the clarke‘s error grid analysis. Above 96.8% out of 125 numbers of tested samples lies in the clinically correct decision range where as less than 3.2% in the clinically uncritical decision region. Figure 8 depicts Parke‘s error grid analysis by testing the CBGM and the PBGM values into the analytic model. This analysis depicts the result values are mostly in A (clinically correct decisions) and a very-few ($> \, 5$) in B (clinically uncritical decisions).

**Figure 9:**
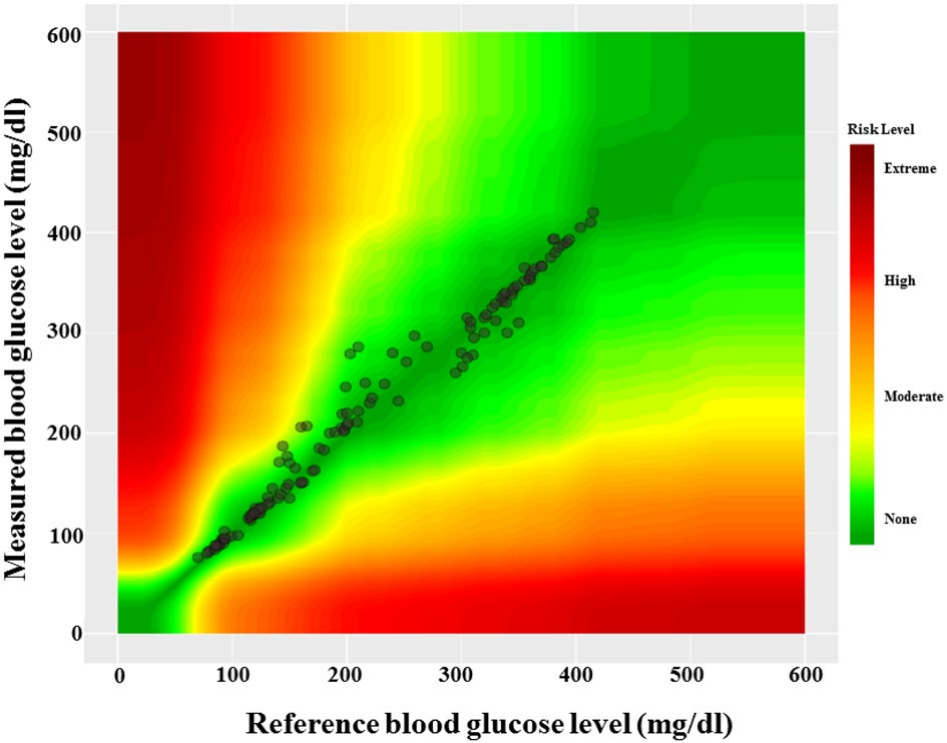
Surveillance error grid analysis for PBGM.

**Figure 10:**
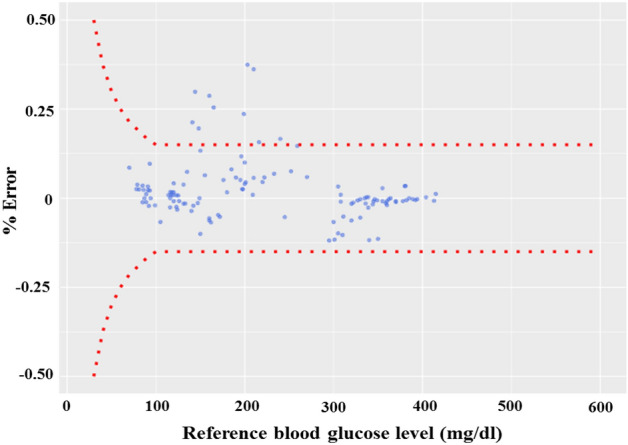
Modified Bland–Altman plot for blood glucometer error surveillance.

As per recent development, the surveillance error grid analysis (SEG) including modified Bland–Altman plot for the system surveillance is carried out. Figure 9 denotes the SEG analysis, where the total no of 125 samples are taken for SEG testing. Out of 125 number of samples, 121 (96.8%) samples are in green zone (risk factor range: 0–0.5%, no risk) and 4 (3.2%) samples are in light green zone (risk factor range: > 0.5–1.0%, slight lower risk). The modified Bland–Altman plot analyzes regression quality by comparing the difference between two measures to their average. The modified Bland–Altman plot is also named as ‘Tukey mean-difference plot’. The deviation of tested data values from the reference point is plotted in Fig. 10 and found very less marginal error. The mean relative difference (Bias), mean absolute relative difference (MARD) and standard relative difference between CBGM and PBGM are 2.2%, 5.3% and 8.3% respectively.

The classification of the blood glucose levels are predominantly described worldwide. Number of tested samples ($^{*}$n) from CBGM and PBGM are chosen as per noraml, prediabetes and diabetes level condition. Table 1 describes the mean ($\mu$) and standard deviations ($\sigma$) of their individual classified ranges. The individual NCC of the sample pairs (17, 39, 16, 14, 95 and 71) are 0.9691, 0.9835, 0.9662, 0.9673, 0.9758 and 0.9665, where as the overall NCC of the samples (125 nos) is 0.9859. Thus indicates the accuracy of individual classifications lie above 96% where as the total accuracy of the PBGM is 98%. Before taking blood inside the testing chamber, the chamber has been cleaned by distilled water. After that by considering distilled water in the chamber, the test run has been carried out for approximately 60 to 90 s and output voltage has been noted. Such type of cleaning and repeating the test run using distilled water has been continued till output voltage reading of $19\pm 0.5$ mV is achieved. Once the system achieved the base reading of $19\pm 0.5$ mV, the system is now ready for blood glucose level measurement. From the above experimental observation it is concluded that, the proposed system holds good for periodically measurement of blood glucose in animal (goat) blood. This will help us to carry out further research in in-vivo condition and on suitability, this measurement system will be applied to human blood to obtain the glucose variation periodically with a short span of time.

**Table 1:** Blood glucose level classification with mean ($\mu$), standard deviation ($\sigma$) of CBGM and PBGM.

Blood glucose classification	Fasting blood glucose levels (mg/dl)^43^	$\mu \pm \sigma$		Post meal blood glucose levels (mg/dl)^43^	$\mu \pm \sigma$	
		CBGM	PBGM		CBGM	PBGM
Normal	70–100 (n = 17)	87.9 ± 8	88.7 ± 6	70–140 (n = 39)	108.0 ± 20	108.1 ± 18
Prediabetes	101–125 (n = 16)	119.7 ± 10	119.5 ± 6	141–200 (n = 14)	159.9 ± 15	168.6 ± 16
Diabetes	125–above (n = 95)	261.5 ± 91	265.3 ± 86	200–above (n = 71)	300.0 ± 72	303.8 ± 63

## Conclusion

To quantify the glucose levels in the blood medium, a system based on ultrasonic MEMS transceivers and the RVM probabilistic model has been proposed. This is a type of in-vitro experiment. This low-cost system can measure the patient’s glucose level on a regular basis as needed. The experimental result values, which include various error grid analysis reports, depict the system’s robustness. CBGM is used to validate the system. The NCC of 0.9859 indicates that there is no statistically significant difference greater than 0.05 between CBGM and PBGM. The proposed system can detect glucose levels as high as 450 mg/dl.

## Data Availability

All data generated or analysed during this study are included in this version of the manuscript.
